# Torsional Vibration Analysis Using Rotational Laser Vibrometers

**DOI:** 10.3390/s24061788

**Published:** 2024-03-10

**Authors:** Steven Chatterton, Ludovico Dassi, Edoardo Gheller, Tommaso Ghisi, Andrea Vania, Paolo Pennacchi

**Affiliations:** Department of Mechanical Engineering, Politecnico di Milano, Via La Masa 1, 20156 Milan, Italy; ludovico.dassi@polimi.it (L.D.); edoardo.gheller@polimi.it (E.G.); tommasoghisi5@gmail.com (T.G.); andrea.vania@polimi.it (A.V.); paolo.pennacchi@polimi.it (P.P.)

**Keywords:** torsional vibration, rotational laser vibrometer, dynamic model, calibration

## Abstract

Torsional vibration is a critical phenomenon in rotor dynamics. It consists of an oscillating movement of the shaft and causes failures in multiple oscillating fields of application. This type of vibration is more difficult to measure than lateral vibration. Torsional vibrometers are generally invasive and require a complicated setup, as well as being inconvenient for field measurements. One of the most reliable, non-invasive, and transportable measuring techniques involves the laser torsional vibrometer. For this research, two laser heads with different measurement capabilities were utilized. An experimental test rig was used to perform a relative calibration of the two laser vibrometers. The frequency of the acting force and the rotation speed of the shaft vary in the same range, which is commonly found in rotating machines. Finally, experimental measurements of torsional vibrations using laser vibrometers were compared with numerical results from a 1D finite element model of the same test rig. The main outcome of this paper is the definition of a reliable measuring procedure to exploit two laser vibrometers for detecting torsional mode-shapes and natural frequencies on real machines. The relative calibration of two different measuring heads is described in detail, and the procedure was fundamental to properly correlate measuring signals in two machine sections. A good correspondence between the numerical and experimental results was found.

## 1. Introduction

Torsional vibration is the periodic oscillation of angular position between two shaft sections that can be observed in rotors. It can be induced by an oscillating torque applied to the shaft but also by shaft assembly errors such as faulty bearings, transmissions, and other common phenomena of rotor dynamics. When torsional vibration occurs, the stress state of the rotating parts changes periodically, and this vibration can have a sufficiently high intensity to cause torsional fatigue phenomena in the rotating shaft. Shaft fatigue caused by torsional vibration stresses accumulates continuously. After reaching a certain level, cracks and notches form on the shaft, which can lead to shaft fracture. Fractures due to torsional fatigue are oriented at forty-five degrees to the axis of the rotating shaft. In rotating shafts these fractures are often located at hubs or couplings. Therefore, measuring and monitoring torsional vibration is important for rotor safety. Meanwhile, the torsional vibrometers are more sensitive to fault signals and they have a lower signal-to-noise ratio compared to lateral vibrometers [1]. Torsional vibrations are critical for various engineering fields, such as power production plants [2,3]. Typical examples of sources of torsional vibration are:

■ Electric motor/generator defects due to electric imbalances and short circuit defects [4];
■ Pump, compressor, turbine, and fan defects cause torsional excitations at an equal or multiples of the blade passing frequency [5,6];
■ Gear drives, where elastic deformation and defective tooth surfaces can cause torsional oscillation;
■ Cardan joints in which the driven shaft is submitted to an oscillation of doubled frequency with respect to the drive one.

In addition to these causes, the misalignment of the motor also causes torsional vibrations: either due to the periodic torque change caused by the abnormal oscillation of the axis of revolution or friction and possible contact between static and rotating parts.

As with lateral vibrations, any mechanical system in the design phase must be considered to have a safety margin in its operating range with respect to its natural torsional frequencies. The frequency safety margin represents the distance between the frequency of the torques applied to the system and the system torsional resonance frequency. Usually, lateral and torsional vibrations are two independent phenomena, but in some special cases, even lateral vibrations are sources of torsional vibrations and vice versa. This is the case when the same source, as a mass unbalance, causes both lateral and torsional excitation [7]. The presence of gear transmissions also couples the two different vibrations, which both change the point of contact between the teeth and change the lateral and torsional stiffness characteristics of the transmission. The development of torsional vibration from lateral vibrations is negligible compared to other vibration sources.

Various measurement techniques are available to catch torsional vibrations. As suggested in [8], the best sensor can be selected for each individual case based on the physical quantity to be measured, the type of analysis, the accessibility of the shaft, the ease of instrumentation, and the required accuracy.

Measuring instruments for torsional vibrations are generally rarely used in comparison to those ones for lateral vibrations. This happens because lateral vibrations are easily measurable and are a source of noise and vibrations that are also transmitted to the foundations. For this reason, only instruments for measuring lateral vibrations are usually installed in most rotors. Torsional vibration measurement is much more expensive and complicated to use. They can be classified into direct and indirect methods. The former technique relies on the direct measurement of the relative angular position between two different sections, requiring the calculation of the time trend of the torsional or angular deformation in the sections of interest. Instruments in this family are linear accelerometers and double-beam laser interferometers. In the first case, two linear accelerometers are fixed face-to-face on the rotating shaft, measuring tangential accelerations. Since they have opposite directions in their rotating systems, any translational acceleration of the shaft is canceled out by taking the average of both accelerometer signals. The torsional vibration is obtained by the double integration of the angular acceleration. Its many shortcomings do not make it a widely used instrument, except in cases where the reliable measurement of torsional vibrations at very high frequencies is needed or when both lateral and torsional vibrations need to be measured with the same instruments [9].

Indirect methods, on the other hand, derive torsional vibrations from other quantities, such as surface stresses. Strain gauges, optical sensors, incremental encoders, and magnetic pick-ups are the main instruments of this family.

Strain gauges are used to measure strain on a surface to determine the internal stress of a material. With this method it is possible to determine the shear deformation of the shaft and consequently calculate the torque in the shaft. When a shaft is subjected to torsion, principal normal stresses occur at an angle of ±45° to the cylindrical planes [10,11].

Optical encoder is a transducer commonly used to measure the angular position of the shaft and widely used in motor control applications [12]. The signal is discrete, and the resolution is rather limited and depends on the number of signs per revolution. This method is very reliable, but only for low frequencies.

Magnetic pickups detect changes in the magnetic field or magnetic flux, usually resulting from the passage of metal teeth through the sensor. However, these sensors are very simple, non-invasive and can withstand suboptimal environmental conditions [13].

The other type of sensor is an optical sensor used on a zebra stripe or zebra disk. Usually, zebra tape introduces a significant error where the two sides of the tape overlap. The instrumentation is economical and simple because the sensor is fixed on a static component. A good quality optical sensor allows the measurement of a high pulse frequency but must be mounted very close to the rotating shaft [14]. Lateral shaft vibrations are a problem both because of the proximity of the sensor to the shaft and because, by changing the relative displacement between the sensor and the shaft, they reduce the quality of the signal and can cause errors in the measurement of torsional vibrations.

Rotational laser vibrometers (RLV) are optical instruments that exploit the properties of a laser to non-invasively measure the torsional vibrations of rotating parts. They can produce an independent measurement of lateral or axial translations and their measurement is independent of the shape of the shaft. Furthermore, a surface with adequate reflective characteristics is required, which can be obtained through the simple application of adhesive tapes or sprays and good alignment with the shaft. The improvement of the reflective characteristics of the surface also allows the use of low power lasers, making the product less dangerous, lighter, and, therefore, transportable. This makes it an ideal technological solution for field measurements. The new models move in the direction of a smaller footprint and similar measurement characteristics [15,16,17]. The optical measurement principle is based on laser interferometry. Angular velocity is calculated by measuring two parallel components of translational velocity. The system consists of two interferometers and two parallel measurement beams. The two speeds in the backscattered rays produce two Doppler frequencies [13]. The sum of these two frequencies gives the resulting Doppler frequency which depends on the separation distance of the laser, the wavelength of the laser, and the angular velocity of the shaft. Torsional vibration is the fluctuating part of the continuous-time voltage signal of the angular velocity [18]. Measuring the angular velocity fluctuation on two different sections of the shaft allows the actual torque and deformation of the shaft to be evaluated. With the measurement of only one section, it is not possible to discriminate the angular velocity fluctuation caused by the rigid motion of the shaft from the real torsion of the shaft.

In the existing literature, the torsional vibration analysis of real rotating machines is a common assessment procedure. It is used for both the monitoring of faulty machines [6,19,20] and the design of innovative torsional dampers to be applied on long shafts lines [21,22]. Usually strain gauges and phonic wheels are used for experimental measurements [23,24], but they require an invasive and dedicated set-up. Bell et al. [16] used laser vibratomes in real-world applications but they limited the measurements to only one section of the machine.

The innovative aspect of this research activity is to firstly consider two independent laser vibrometers in order to experimentally measure the torsional modes on a dedicated test rig. The first issue to be addressed is the definition of a calibration procedure calibration of two measuring units with distinct technology. This important step highlights the differences of the two measuring units and allows the calculation of the correction terms, which is fundamental to combine the two signals.

An experimental test rig is designed and assembled to carry out the experimental measurement. The test rig consists of a thin shaft; inertial disks, which are also the measurement sections; the transmission; and two motors—one to control the rotation speed and one to control the torque. After the relative calibration of the two instruments, the torsional behavior of the test rig was measured experimentally and compared with the predicted torsional behavior obtained by a 1D finite element model. An incremental optical encoder was mounted on the shaft for a further comparison with laser vibrometer measurements. This comparison is useful because it highlights the many advantages of rotational laser vibrometers over optical encoders, which are still the most widely used instruments for measuring torsional vibrations.

## 2. Experimental Setup

**Figure 1:**
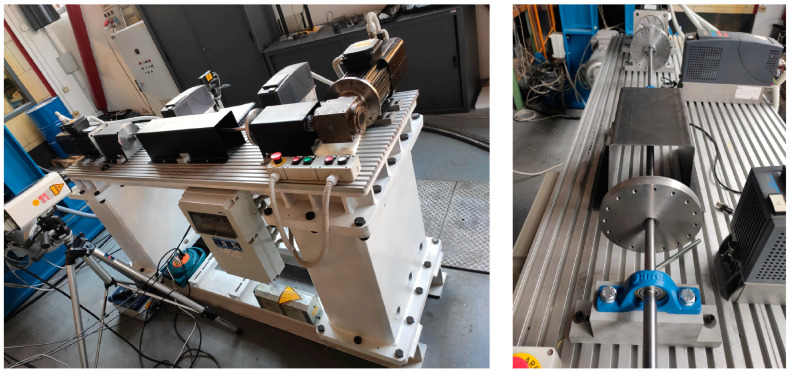
Test rig for torsional measurements.

**Figure 2:**
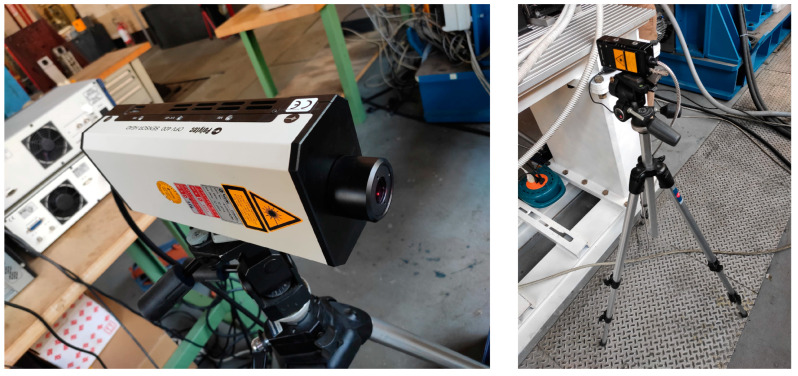
Laser vibrometers: Polytec OFV-4000 (**left**) and RLV-5000 (**right**).

The test rig is shown in Figure 1, it consists of a thin shaft 15 mm in diameter and 1300 mm long, supported by two ball bearing units. An additional ball bearing unit is installed in the center span of the shaft to avoid any interference of shaft bending vibrations on the measurements. Two disks with diameters of 150 mm are mounted on the shaft using locking devices. On the drive side, the shaft is connected to a 90° single-stage reducer with a reduction ratio of τ = 2 and to an AC motor via a rigid torsional coupling. This motor is speed-controlled. The non-driven side of the shaft is connected to a brake motor via a rigid torsional coupling. The brake motor is used to apply pulsating torque to the system. The frequency response of the test rig can be changed by installing additional disks in order to increase the inertia of the system. The disks are the only sections where the two laser vibrometers, Polytec (Baden-Württemberg, Germany) OFV-4000 and RLV-5000, can be used (Figure 2). The main reason for this is that the distance of the laser beams (8 mm) is too high compared to the shaft diameter (15 mm). The measurement could be negatively affected. A reflective adhesive tape is applied to the cylindrical surface of the disks to increase the signal-to-noise ratio in the measurements.

The two laser heads have a high-quality interferometer configuration, and the laser source is a helium–neon (He-Ne) gas mixture, with a wavelength of λ = 633 nm. The two laser interferometers operate with a low output power (1–3 mW) and without any risk for the operator. The dynamic acquisition of rotational vibrations is possible in the frequency range from 0.5 Hz to 10 kHz, thus covering even the most demanding measurement tasks. The measurement accuracy is independent of the direction of rotation.

The two laser vibrometers are designed to acquire the dynamic part of the rotation speed. Of secondary importance is the acquisition of the average rotation speed of the object to be measured.

The resolution can be highly affected by the irregularities on the measuring surface, due to the speckled nature of the reflected light. The largest spectral components of this noise lie naturally in the harmonics of the rotational speed frequency, so unfortunately, the worst resolution is achieved at these frequencies. The amount of disturbance caused by the speckle noise is greatly dependent on the surface quality and on the angular speed. Faulty patches in the shaft circumference, such as grooves or chips in the retroreflective film, cause big interferences.

The data for the two laser vibrometers are listed in Table 1.

## 3. Torsional Model

**Figure 3:**
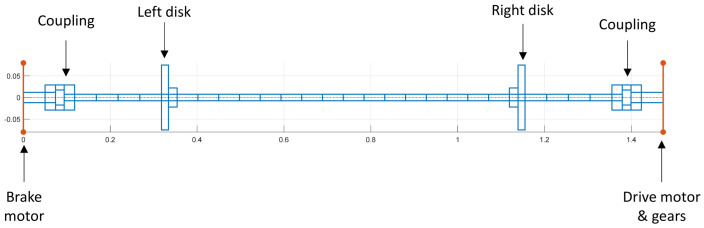
FEM of the rotor system.

A 1D finite element model of the shaft was developed to model only the torsional dynamics of the test rig. The primary elements are Bernoulli beams, and each node has only one degree of freedom, namely the absolute rotation of the shaft section. The mass and the stiffness of each element were calculated according to Equation (2). The numerical model was implemented and solved using Matlab^®^ 2023 language. The torsional model shown in Figure 3 includes the portions of the shaft, two disks (the left one and the right one), two motors (brake and the speed motors), transmission, and two rigid couplings.

The motors and gears are considered lumped disks placed at the ends of the shaft with a reduced mass moment of inertia due to the transmission ratio of the gearbox.

The equation of motion of the model is as follows:

(1) $$\left[M\right]\ddot{\theta }+\left[K\right]\theta =T$$

where θ is the vector of angular rotation of the nodes, T is the vector of applied torques of the two motors, and $\left[M\right]$ and $\left[K\right]$ are the mass and the stiffness matrices, respectively, obtained by assembling the mass matrix $\left[M_{i}\right]$ and stiffness matrix $\left[K_{i}\right]$ of the *i*-th element:

(2) $$\left[M_{i}\right]=\frac{\rho_{i}I_{i}L_{i}}{6}\left[\begin{array}{cc} 2 & 1\\ 1 & 2 \end{array}\right]\;\left[K_{i}\right]=\frac{G_{i}I_{i}}{L_{i}}\left[\begin{array}{cc} 1 & -1\\ -1 & 1 \end{array}\right]$$

$\rho_{i}$ is the mass density, $L_{i}$ is the length, $I_{i}$ is the area polar moment of inertia of cross-section for the *i*-th beam element, and G is the tangential modulus of the material.

The one-dimensional model has been selected for its simplicity; moreover, the model has been widely used, and it is confirmed to be reliable for studying rotating machines [20,21]. The geometry of the test rig is generally simple and main parts can be easily discretized by cylindrical elements with homogeneous diameter and material properties. The more complex components (angular transmission and motor rotor parts) are simplified and considered lamped masses.

An important simplification hypothesis of the considered model is the neglect of the rotational damping. From a physical point of view, the considered test rig has very low damping levels, the mail components (ball bearings, mechanical transmission, and couplings) were selected to be fully rigid, and the resulting system is stiff. The low damping level is also confirmed in the existing literature, and it is usually considered an issue for real rotating machines [6,19,20]. Moreover, the estimation of the system damping is an important aspect when calculating the torsional stress in the shaft line and fatigue assessment. For the current analysis, low levels of damping only have a marginal effect on the natural frequencies of the system and the related mode shapes.

**Figure 4:**
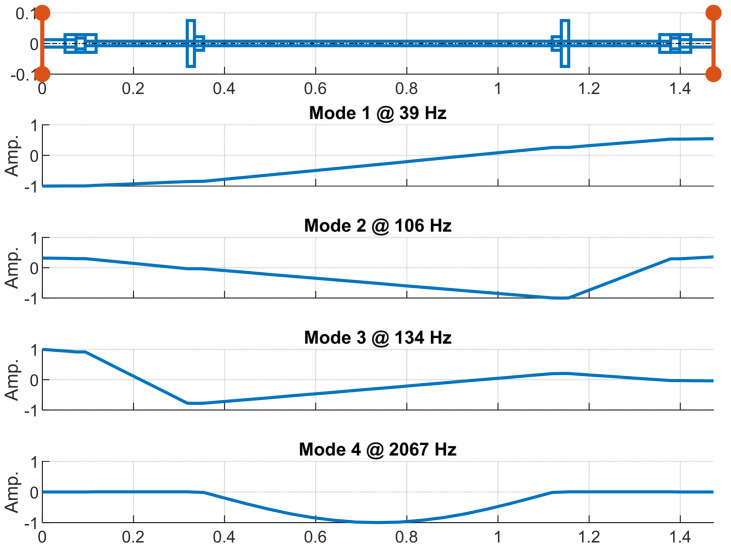
Vibrating modes of the rotor system FEM.

Once the model is implemented, an eigenvalue analysis is then performed on the homogeneous Equation (1) to obtain eigenfrequencies and vibrating modes. The first four modes of vibration are shown in Figure 4 along with the eigenfrequencies: 39 Hz, 106 Hz, 134 Hz, and 2067 Hz. The first three eigenfrequencies are within the measurement range of the vibrometers.

The first and third modes of vibration have the maximum displacement at the brake motor node. These modes are also the most excited by the oscillating torque in agreement with the experimental results. The value of the first eigenfrequency falls in the 30–50 Hz range, where the mechanical reducer generates high noise in the experimental tests. The second mode of vibration is slightly excited in the experimental tests as confirmed by the low amplitude of the vibrating mode at the node of the brake motor, while the value of the second natural frequency corresponds well with the experimental value.

## 4. Calibration of Laser Vibrometers

Calibration between the two laser vibrometers is necessary, since the two measuring systems have different characteristics and different output responses.

**Figure 5:**
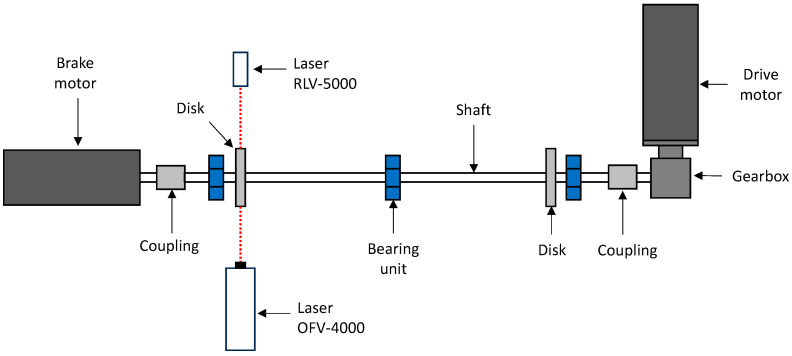
Layout for the calibration of the laser vibrometers.

In general, a “calibration” refers to a situation in which all but one of the inputs are held at constant values. Then, this input is varied over a range, called the “calibration range”. The input–output relationships thus calculated constitute a static calibration valid in the conditions of all other inputs. This procedure can be repeated, varying each input deemed of interest from time to time and thus developing a large number of static input–output relationships [25]. The simplest way to investigate this relationship is to have the two instruments measure the same quantity. In the case of torsional vibration, it is sufficient to position the two instruments in the same section as close as possible to the excitation (the brake motor) to have a clear signal and a high signal/noise ratio. The simplest methodology is to measure the angular speed vibration on the same rigid disk. The rotational speed of the shaft and the frequency of the oscillating torque of the brake motor are changed, as shown in Figure 5. A constant positive torque is also applied in order to avoid the gear noise due to the periodic change of torque direction.

Ideally, the instrument signals should have the same phase and amplitude. A minus sign has been added to the second signal to account for the opposite installation of the laser head with respect to the direction of rotation of the shaft. By considering a harmonic torque excitation at frequency f, the measurements of the two vibrometers are as follows:

(3) $$\begin{array}{l} d\omega_{1}=A_{1}\cdot \cos\left(2\pi f\cdot t+\varphi_{1}\right)\\ d\omega_{2}=A_{2}\cdot \cos\left(2\pi f\cdot t+\varphi_{2}\right) \end{array}$$

The sensitivity selected for the two laser vibrometers is 100°/s/V, and the resolution of the acquisition system is 24 bits. In general, the lowest values of dω are recorded when the excitation frequency is 200 Hz, and they are close to 10 °/s. Therefore, the relative error of the laser vibrometer measurement is 5.9 × 10^−6^. The excitation current is measured by the motor with a resolution of 16 bits. The sensitivity of the current measurement is 1 A/V. The minimum current amplitude is measured when the excitation frequency is 200 Hz and is close to 0.4 A. Therefore, the relative error of the current measurement is 4.3 × 10^−4^. Multiple acquisitions were performed for each test condition and the average was used in the calculation of the calibration parameters and of the transfer functions. The good repeatability of the measurements was observed.

The calibration gives the amplitude difference error $\Delta A_{c}$, the phase difference $\Delta \varphi_{c}$, and the time delay $\Delta T_{c}$ between the two instruments, as follows:

(4) $$\begin{array}{l} \Delta A_{c}=\left(A_{2}-A_{1}\right)/A_{1}\\ \Delta \varphi_{c}=\varphi_{2}-\varphi_{1}\\ \Delta T_{c}=\frac{\Delta \varphi_{c}}{2\pi f_{0}} \end{array}$$

**Figure 6:**
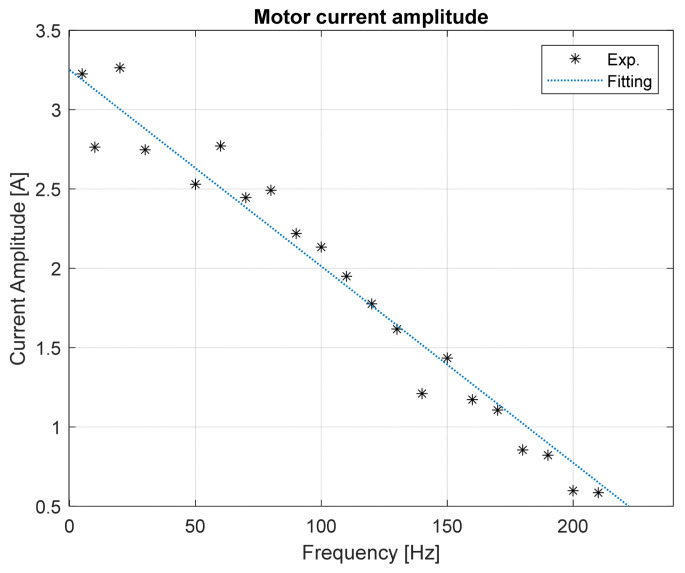
Motor current amplitude as a function of the torque frequency.

In these tests, the amplitude of the excitation torque applied to the brake motor decreases with the increase in the excitation frequency, as shown in Figure 6, for a rotation speed of 500 rpm, while it remains constant for a fixed excitation frequency by varying the speed of the shaft.

**Figure 7:**
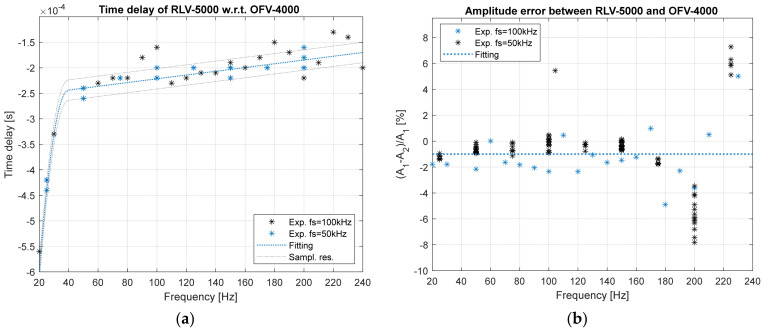
(**a**) Time delay between laser vibrometers as a function of the torque frequency; (**b**) amplitude error as a function of the torque frequency.

The time delay between the output of two sensors was obtained by cross-correlating the two signals. The time delay decreases by increasing the torque frequency, as shown in Figure 7a, for a shaft speed of 500 rpm. Dedicated calibration tests have been performed with a higher sampling rate (100 kHz) in order to guarantee a time resolution of 1 × 10^−5^. The delay remains constant at approximately 2 × 10^−4^ s by changing the shaft speed for a fixed torque frequency of 150 Hz. In other words, the vibrometer type RLV-5000 is always delayed in comparison to type OFV-4000.

The signal amplitude error was obtained in the frequency domain, as shown in Figure 7b, as a function of the torque frequency. The dispersion of the experimental points increases with the torque frequency. For torque frequencies up to 200 Hz, there is a dispersion within ±1.5% of the error, but for torque frequencies above 200 Hz, the dispersion becomes ±7%. The increase in relative error is due to the small amplitude of the vibration above 200 Hz. The amplitude of the vibration depends directly on the amplitude of the forcing torque, as shown in Figure 6. Furthermore, the third natural torsional frequency of the rotor is less than 200 Hz. Therefore, the system works in seismographic conditions, causing a further attenuation of torsional vibrations to the given excitation. In other words, if A_1_ and A_2_ are very small, even a small difference causes the larger dispersion of the relative amplitudes.

## 5. Encoder Comparison

**Figure 8:**
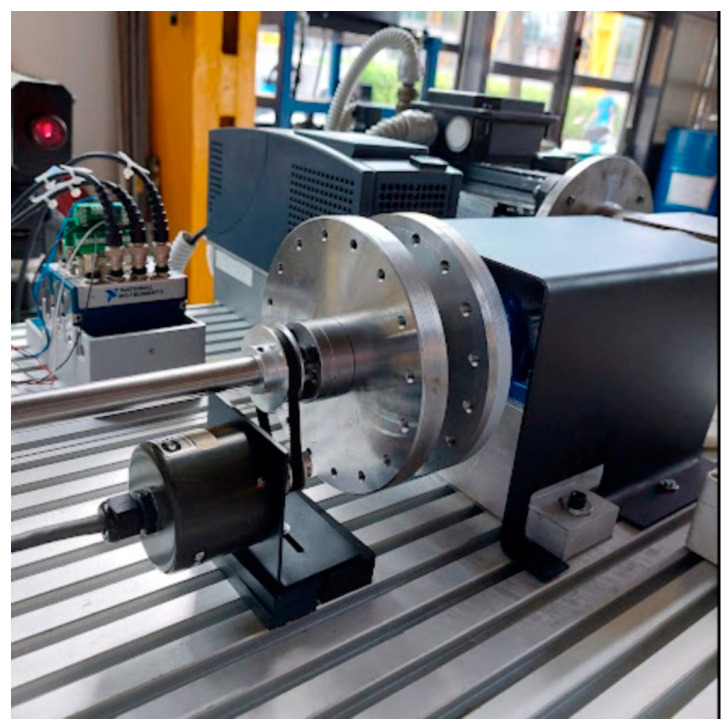
Detail of the encoder installation.

The incremental optical encoder is a conventional instrument for measuring the angular speed and the angular position of a rotating shaft. It is a conventional instrument in the sense that it is quite common and inexpensive compared to laser vibrometers. However, this instrument is invasive, because its installation modifies the inertia of the system and requires a pulley–belt system. So, the set-up of an encoder vibrometer is much more time-consuming and complex compared to that of the rotational laser vibrometer. The pulley–belt encoder connection is elastic; therefore, a phase delay between the rotation of the shafts and encoder can be expected in the experimental measurements. A 4000 counts/rev encoder is mounted on the shaft, close to the disk, as shown in Figure 8.

**Figure 9:**
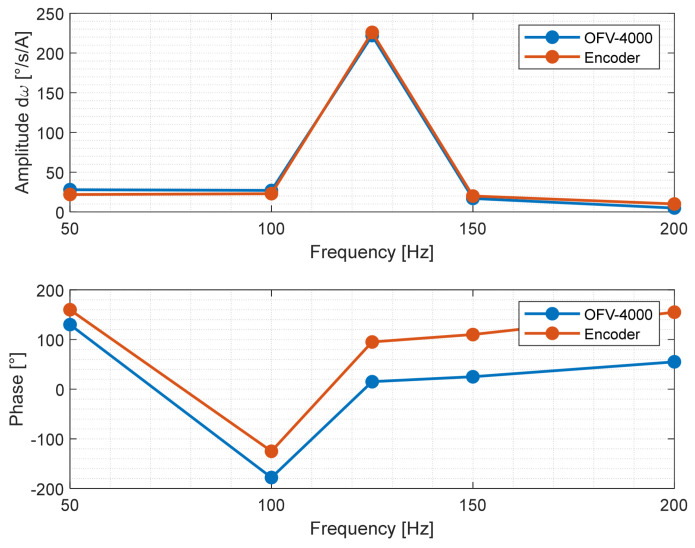
Comparison between laser vibrometer OFV-4000 and encoder measurements.

A measuring comparison was performed between the encoder and the OFV-4000 laser vibrometer. Several values of the frequency of the torque of the brake motor have been considered, while the angular speed of the shaft was kept constant at 500 rpm. The measured frequencies were 50, 100, 125, 150, and 200 Hz. The acquisition time was instead kept at 20 s to maintain the same resolution frequency as the calibration. The encoder signal is digitally filtered with a high-pass filter at a frequency of 5 Hz to obtain the continuous rotational signal. The signal is post-processed to calculate the shaft speed. The spectrum of this function is then calculated by means of a Hamming window. The amplitude and phase for the encoder is compensated by taking into account the different diameters of the two pulleys. The comparison is shown in Figure 9, where the amplitudes show good agreement, whereas a fairly constant delay occurs on phase component.

## 6. Torsional Vibration Measurements

**Figure 10:**
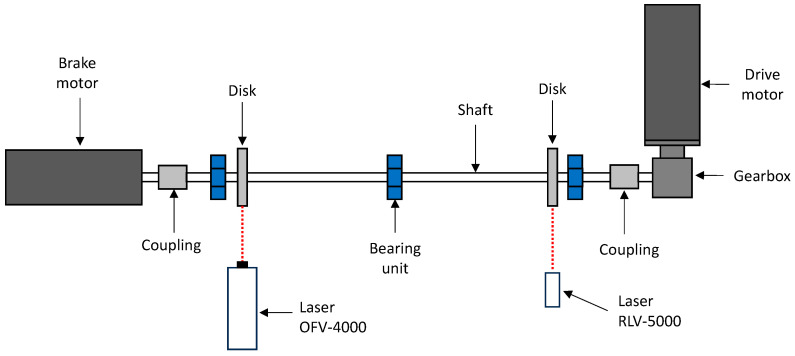
Layout for the torsional vibration measurements.

The measurement of the torsional vibration is conducted with two laser vibrometers positioned at two different sections of the rotating shaft, as shown in Figure 10. This arrangement allows us to estimate the torsional stress in the shaft, which depends on the difference in angular rotation between the two sections. After taking measurements with this configuration, the positions of the two vibrometers are reversed and the measurements are repeated under identical conditions. By switching the two instruments, it is possible to directly compare the two vibrometers, and thus the accuracy of the calibration can easily be verified.

The tests were performed by changing the torque frequency from 20 to 240 Hz with steps of 5 Hz. Measurements were repeated three times to ensure reproducibility. The test was not performed in the frequency range of 30–50 Hz due to the loud noises and vibration of the reducer caused by the first system’s torsional vibrating mode. The angular speed was maintained at a constant of 500 rpm. Measurements were taken at a sampling frequency of 25 kHz with a window time of 20 s in order to maintain a good frequency resolution in the signal spectrum. Finally, it is possible to define the angular velocity difference between the two disks as follows:

(5) $$\begin{array}{l} d\omega_{1,L}=A_{1,L}\cdot \cos\left(2\pi f\cdot t+\varphi_{1,L}\right)\\ d\omega_{2,L}=A_{2,L}\cdot \cos\left(2\pi f\cdot t+\varphi_{2,L}\right)\\ d\omega_{1,R}=A_{1,R}\cdot \cos\left(2\pi f\cdot t+\varphi_{1,R}\right)\\ d\omega_{2,R}=A_{2,R}\cdot \cos\left(2\pi f\cdot t+\varphi_{2,R}\right) \end{array}$$

where $d\omega_{k,i}$ is the speed fluctuation measured by the *k*-th laser vibrometer (*k* = 1 for OFV-4000; *k* = 2 for RLV-5000) on the *i*-th disk (*i* = *L* for the left disk; *i* = *R* for the right disk). Amplitudes $A_{k,i}$ and phases $\varphi_{k,i}$ depend on the torque frequency.

To correctly use the two instruments in combination with these measurements, the measurement must be corrected with the calibration parameters $\Delta A_{c}$ and $\Delta \varphi_{c}$ of Equation (4):

(6) $$d\omega_{2,L}=A_{2,L}\cdot \left(1+\Delta A_{c}\right)\cdot \cos\left(2\pi f\cdot t+\varphi_{2,L}-\Delta \varphi_{c}\right)\;d\omega_{2,R}=A_{2,R}\cdot \left(1+\Delta A_{c}\right)\cdot \cos\left(2\pi f\cdot t+\varphi_{2,R}-\Delta \varphi_{c}\right)$$

**Figure 11:**
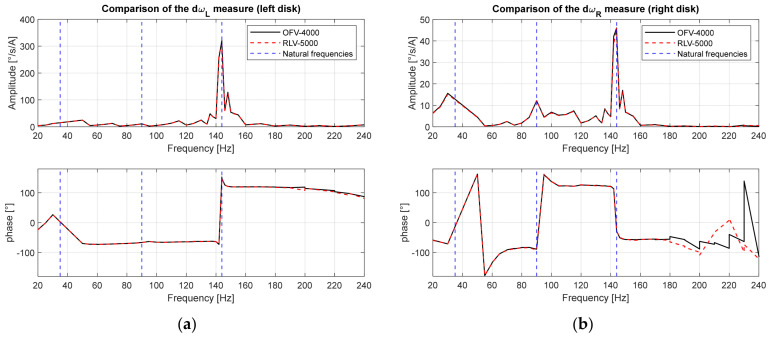
Transfer function of disk response: (**a**) left disk; (**b**) right disk.

The transfer functions between the brake motor current, and the vibrations of the two disks measured with the two laser vibrometers are shown in Figure 11. The vibrations on the same disk measured by the two laser vibrometers obtained by switching their positions are very similar. This result confirms the accuracy of the calibration procedure. A discrepancy occurs on the phase measurement at high frequency (>200 Hz) for the right disk (Figure 11b). This is due to the low exciting torque (motor current) of the brake motor, as confirmed by the low amplitudes at these frequencies.

In Figure 11, it is possible to highlight the presence of three main system resonances placed at 30–50 Hz, 90–100 Hz, and 140–150 Hz. In particular, the second resonance is detected only on the right disk, as expected by the nodal position of the left disk for the second vibrating mode (Figure 4).

To investigate the level of stress to which the shaft segment between the two disks is subjected, the angular vibration between the two sections must be evaluated. The angular velocity difference between the two sections of the disks can be computed as follows:

(7) $$\Delta \omega =d\omega_{1,L}-d\omega_{2,R}$$

**Figure 12:**
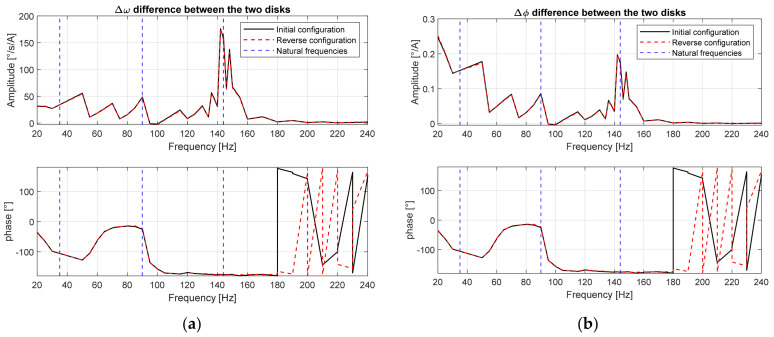
Transfer function of vibration difference between the two disks: (**a**) angular velocity; (**b**) angular displacement.

The spectrum of Δω is shown in Figure 12a, in which the third natural frequency has the largest amplitudes for angular velocity vibration, while the amplitudes at the other two natural frequencies have small amplitudes. The phase between the two disks at their respective frequencies depends on the shape of their mode of the vibrations. Once the spectrum of rotational speed vibration is obtained, it is easy to obtain the spectrum of the angular displacement Δϕ between the two disks as well, as shown in Figure 12b. At low frequencies, the first natural frequency causes significantly greater angular displacements than the other two natural frequencies.

## 7. Conclusions

Laser torsional vibrometers have been proven to be suitable instruments for the on-field measurements of torsional vibration. They are easily transportable, mountable, non-invasive, and quite simple to use. These instruments are ideal for detecting high-frequency vibrations. They require a proper calibration, when used in combination, to measure torsional vibrations between two distinct sections. The amplitude difference and the phase delay between the two lasers was computed by measuring the same section of the assembled test bench. This test rig allows for the adjustment of the oscillating torque frequency, which is found to be the primary variable, influencing the measurement quality of laser vibrometers. Contrarily, it was observed that angular velocity has a minimal influence on the measurements of the two instruments.

The mathematical quantification of the differences between the two measurements can be achieved using basic tools from signal analysis theory, including cross-correlation and Fourier transform. In the postprocessing phase, the main issues are related to the high sampling rate, required for the precise calculation of the time delay, and the high number of measurements, to be taken to obtain consistent data (several repeated measurements are required for each state of the system). One of the main outcomes of this work is the demonstration of the accuracy of phase shift calculations, suggesting that, in the future, focusing solely on the frequency domain will be feasible by limiting the sampling frequency and reducing the size of the data considered. Several interesting future developments in this research area pertain to improving the assembled test rig. Indeed, it displays a high level of adaptability in the ability to change the types and positions of the components. Another noteworthy application of the test rig involves the various gear transmissions and their impacts on torsional vibrations. Other measuring instruments could be mounted to compare the quality of measurements of the different types of vibrometers.

## Figures and Tables

**Table 1:** Laser vibrometer specifications.

Vibrometers	RLV-5000	OFV-4000
Laser Type	HeNe, 1 mW	HeNe, 3 mW
Wavelength	633 nm	633 nm
Beam Separation $d_{l}$	7.5 mm	8 mm
Standoff distance	600±50 mm	400±50 mm
dω slope	10−100−1000−6000°/s/V	10−100−1000−6000°/s/V
dθ slope	0.01−1−10°/V	0.01−1−10°/V
RPM slope	1000 rpm/V	2000 rpm/V
Voltage Range	±10 V	±10 V

## Data Availability

The datasets presented in this article are not readily available due to technical limitations.
